# Do we rely on good-enough processing in reading under auditory and visual noise?

**DOI:** 10.1371/journal.pone.0277429

**Published:** 2023-01-24

**Authors:** Nina Zdorova, Svetlana Malyutina, Anna Laurinavichyute, Anastasiia Kaprielova, Anastasia Ziubanova, Anastasiya Lopukhina

**Affiliations:** 1 Center for Language and Brain, HSE University, Moscow, Russia; 2 Institute of Linguistics, Russian Academy of Sciences, Moscow, Russia; 3 Department of Linguistics, University of Potsdam, Potsdam, Germany; Tohoku University, JAPAN

## Abstract

Noise, as part of real-life communication flow, degrades the quality of linguistic input and affects language processing. According to predictions of the noisy-channel and good-enough processing models, noise should make comprehenders rely more on word-level semantics instead of actual syntactic relations. However, empirical evidence supporting this prediction is still lacking. For the first time, we investigated whether auditory (three-talker babble) and visual (short idioms appearing next to a target sentence on the screen) noise would trigger greater reliance on semantics and make readers of Russian sentences process the sentences superficially. Our findings suggest that, although Russian speakers generally relied on semantics in sentence comprehension, neither auditory nor visual noise increased this reliance. The only effect of noise on semantic processing was found in reading speed under auditory noise measured by first fixation duration: only without noise, the semantically implausible sentences were read slower than semantically plausible ones. These results do not support the predictions of the study based on the noisy-channel and good-enough processing models, which is discussed in light of the methodological differences among the studies of noise and their possible limitations.

## Introduction

Within theory of communication, noise is considered an inevitable feature of communication flow [1]. Noise can broadly be defined as any disturbance of the communication channel per se, or any additional signal that interferes with the target signal (e.g., advertisement posts on a website, other talkers around, etc.). Noise can also be classified as internal (caused by aging, diseases, or brain damage) or external (produced by environmental conditions) [2]. External noise may have different modality (auditory, visual), matching or mismatching the modality of the target signal. In this regard, a street background noise for people who are talking is a modality match, whereas a street background noise for a person who is reading a book is a modality mismatch. In our study, noise refers to an external linguistic signal in either modality that is presented simultaneously with the target signal.

Overall, previous studies reported a detrimental effect of both auditory and visual noise on reading fluency and comprehension, though their results varied. Eye-movement studies found longer fixations, a greater number of regressions and hence, longer reading time under intelligible [3–5] and unintelligible background speech [6]; under non-linguistic visual noise, such as certain font type or blurred script ([7, 8] in older adults only); and under linguistic visual noise with short phrases appearing on the screen together with target sentences [9]. While noise influences reading fluency, it is unclear whether noise affects reading comprehension: visual noise studied in [9] did not impede readers’ comprehension but auditory noise showed both detrimental and null effects on comprehension accuracy across studies. For instance, comprehension was hampered by background unintelligible speech and music with lyrics that were in the same language as the reading task in [10] and under non-preferred background music in [11]. However, comprehension was not affected when the noise was intelligible speech in native and foreign languages in [5], under bar-type noise such as music and voices in [11, 12].

So far, none of the studies exploring the influence of noise evaluated it in the framework of the language processing theories, e.g., the noisy-channel model [13–15]. According to it, under noise, comprehenders process linguistic input superficially and rely on plausible semantic representations that are not necessarily syntactically licensed. This is to say, comprehenders are predicted to guess relations between words based on their meaning and to rely on their real-world knowledge instead of grammatical information. Some evidence supporting this prediction was reported in [15], where the rate of literal interpretations of semantically implausible sentences decreased as the noise rate (the percent of semantically implausible fillers among stimuli) increased; in [16], where visual noise led to a greater reliance on sentential context and word predictability; and in [17, 18], where visual noise stimulated a greater reliance on word-level semantics instead of text-level semantics.

Similarly with the noisy-channel account but without an emphasis on noise, the good-enough sentence processing model (e.g., [19, 20]) considers semantic plausibility to be one of the driving factors of sentence comprehension. According to the latter, when comprehenders encounter a sentence, two mechanisms of sentence processing launch simultaneously: an algorithmic, syntactically-based, bottom-up processing and a semantically-based, top-down processing. The semantically-based processing can be completed faster if the interim representation is semantically plausible and meets the real-world knowledge. Hence, comprehenders are likely to save their cognitive resources by abandoning the syntactically-based processing. In this scenario, we come up with a final sentence representation based on semantic relations but not on the actual syntactic relations between words.

The good-enough processing model was tested in semantically implausible passive sentences like *The dog was bitten by the man* (e.g., [21, 22]), for which 32% of participants incorrectly identified the agent of the sentence and 26% incorrectly identified its patient [22], endorsing a semantically plausible interpretation instead of the actual syntactically licensed one. Moreover, readers in an ERP study [23] did not detect typical responses to semantic anomalies even in semantically implausible sentences with canonical word order like *The fox that hunted the poacher stalked through the woods*. Instead, the readers built a sentence representation consistent with their real-world knowledge where the poacher hunted the fox. Hence, experimental evidence showed that a highly plausible semantic scheme triggered by the linguistic input can overrule algorithmic parsing and lead to a representation that is unfaithful to the actual input.

As we see, the noisy-channel account and the good-enough sentence processing model share ideas on the mechanisms of sentence comprehension [24, 25]. They both assume that sentence comprehension is not always derived from a veridical representation of the linguistic input. Both models aim to explain language processing in real communication rather than describing isolated grammatical parsing. They also consider semantic plausibility as a driving factor of sentence comprehension. Finally, both models assume that comprehension may be affected by a processing difficulty, or noise, that can include both environmental noise and cognitive/processing constraints.

In cognitively taxing conditions [26], such as under external noise in the present study, the reliance on semantically-based processing postulated by the good-enough and noisy-channel accounts might increase for the following reason: A parser has to allocate cognitive resources to noise inhibition and is consequently left with less resources for language processing. Under noise, once a semantically-based processing is completed and resulted in a semantically plausible representation, the parser might not have sufficient cognitive resources to pursue algorithmic processing (as they are spent on noise inhibition). Therefore, in the presence of external noise, we can expect more good-enough sentence representations. These representations may not necessarily correspond to the actual representation encoded in the sentence.

Previous studies on noise and reliance on semantic information either tested noise as imperfections in the target signal, such as dynamic visual noise in [17, 18] and syntactic errors in the stimuli in [15], or measured reliance on semantics differently from the concepts of semantic (im)plausibility [16–18]. The present study aimed to test for the first time whether auditory (background babble) and visual (short phrases appearing next to the target sentence) linguistic noise increases reliance on semantic plausibility when reading, implying greater reliance on good-enough sentence processing. We report two eye-tracking while reading experiments run with the same stimuli but different types of noise (auditory or visual) in monolingual Russian adults.

Based on the assumption of greater cognitive load under noise and the partial allocation of cognitive resources to noise inhibition, we expected to see 1) the main effect of noise on fixation durations and on sentence comprehension accuracy (reflecting its general effect on sentence processing difficulty), as well as 2) the interaction between noise and semantic plausibility (reflecting a change in qualitative sentence processing mechanisms). Participants would rely on semantics more under noise conditions than under no-noise conditions, so we expect more comprehension errors in semantically implausible sentences.

The overall effect of noise on performance in the noise condition, as compared to the no-noise condition, could be two-fold. On the one hand, participants could prioritize speed over comprehension. The fast and superficial reading would result in accuracy decrease due to the lower attention to the questions and rather random answers across both plausible and implausible conditions. That would be an accelerating main effect of noise on reading time and a detrimental effect of noise on comprehension accuracy.

On the other hand, participants might prioritize comprehension over speed and read sentences more slowly to compensate for the increased cognitive load. The slow-down in reading might enable them to succeed in sentence comprehension. In fact, eye-tracking studies of reading under noise suggest such a pattern (see [3–6] for auditory noise and [7–9] for visual noise). They showed changes in eye movements while reading under noise, but not necessarily changes in comprehension. A possible analogy with this effect would be the real-life conditions when we are often exposed to some level of noise (street and nature noises, background babbles, pop-up notifications on smartphones, advertisement posts on a TV/computer screen), which enables our cognitive system to adapt and successfully handle language processing under noise without comprehension loss.

Although the precise impact of noise on reading comprehension is difficult to predict, due to the lack of developed theoretical frameworks, we outline the potential mechanisms that might affect reading under noise conditions. Both types of noise should increase cognitive load, which should then disrupt reading. At the same time, the cognitive load might differ depending on the modality mis(match) with the target. Studies of language processing in two modalities simultaneously (e.g., reading a text and listening to it) [27–29] found significant comprehension decrease when participants were exposed to a story in both modalities simultaneously, which could confirm a greater negative impact of auditory noise on reading due to modality mismatch. However, their auditorily presented information was both phonologically and semantically overlapping with the target written information, which makes it hard to disentangle the effect of modality from the effect of overlap. Hence, if comprehension does suffer from noise, we expect it to be hampered either under both auditory and visual noise due to a greater cognitive load; or under auditory noise to a greater extent due to the modality mismatch.

Most importantly, we expect to find a plausibility by noise interaction both in sentence reading and comprehension. Previous studies on noisy input (e.g., [15–18]) showed a greater reliance on superficial, semantically-based processing under noise. Similarly, studies on good-enough sentence processing (e.g., [21–23]) demonstrated greater reliance on semantic relations between words when presented with syntax structures that are difficult to parse. Based on these studies, we hypothesize that external auditory and visual noise would also increase reliance on good-enough processing in our experiments.

Concretely, we expect that in semantically implausible sentences, accuracy will be even lower under noise. However, the opposite pattern is also possible: participants may strategically slow down to counteract external noise. This slow-down would probably enable them to do syntactic reanalysis. In such a case, we expect that the accuracy decrease in semantically implausible sentences would not be modulated by noise.

## Experiment 1 (auditory noise)

### Method

#### Participants

Seventy-one adult monolingual speakers of Russian (38 women; M_age_ = 22 years; SD *=* 4.9; range 20–40; mean years of education = 14, range 11–20) took part in the experiment with auditory noise. All participants reported normal or corrected-to-normal vision and hearing, and no history of neurological, psychiatric or language disorders. None but one participant had an educational background in Linguistics. All participants signed an informed consent form before participation. The study was carried out in accordance with the Declaration of Helsinki and the code of conduct of the American Psychological Association and was approved by the HSE Committee on Interuniversity Surveys and Ethical Assessment of Empirical Research.

#### Apparatus

Eye movements were recorded using the Eyelink 1000+ Desktop mount eye-tracker with a chin rest. Stimuli presentation was programmed in the Experiment Builder software (SR Research Ltd.). Sentences were presented in black font (Ubuntu Mono, 30 pt) on a light-gray background. Participants were seated 92 cm away from a 24” monitor with a 1920 x 1080 pixel resolution.

#### Materials

Experimental items (N = 56) were unambiguous Russian sentences with a participial clause attached to one of the two nouns of the genitive noun phrase (NP). We manipulated the plausibility of the semantic match between the participial clause and attachment site (plausible / implausible). Syntactic attachment site of the participle to either the head noun (high attachment) or the dependent noun (low attachment) in the genitive NP was balanced across stimuli, and was unambiguously signaled by case inflection. We chose this particular sentence structure because previous studies [30–32] reported a processing difficulty for such sentences in Russian native speakers without language deficits, which ensured that the sentences were challenging enough in order to detect plausibility effects. Example experimental items with comprehension questions can be found in Table 1. Conditions (1) and (2) were semantically plausible sentences where the participial clause followed the main clause with high and low syntactic attachment, respectively. Conditions (3) and (4) were semantically implausible sentences where the participial clause also followed the main clause with high and low syntactic attachment. All experimental sentences were followed by a binary-choice comprehension question targeting the attachment site of the participle.

**Table 1:** Example of an experimental item.

Semantic Plausibility		
**Plausible**	**(1)**	*Дима работал с* ***доктором*** *президента*, ***лечащим*** *маленьких детей*. Dima worked with **the doctor (Instr, masc)** of the president (Gen, masc), *who treat-PART* (Instr, masc) small children. *Кто лечил маленьких детей*?*—* ***Доктор*** */ Президент* Who treated small children?—**Doctor** / President
	**(2)**	*Дима работал с доктором* ***президента***, ***управляющего*** *целой страной*. Dima worked with the doctor (Instr, masc) of **the president (Gen, masc)**, *who run-PART* (Gen, masc) an entire country. *Кто управлял целой страной*?*— Доктор /* ***Президент*** Who ran an entire country?—Doctor / **President**
**Implausible**	**(3)**	*Дима работал с* ***доктором*** *президента*, ***управляющим*** *целой страной*. Dima worked with the **doctor (Instr, masc)** of the president (Gen, masc), *who run-PART* (Instr, masc) an entire country. *Кто управлял целой страной*?*—* ***Доктор*** */ Президент* Who ran an entire country?—**Doctor** / President
	**(4)**	*Дима работал с доктором* ***президента***, ***лечащего*** *маленьких детей*. Dima worked with the doctor (Instr, masc) of the **president (Gen, masc)**, *who treat-PART* (Gen, masc) small children. *Кто лечил маленьких детей*?*— Доктор /* ***Президент*** Who treated small children?—Doctor / **President**

Importantly, answers to comprehension questions across conditions had different implications. Namely, semantically plausible conditions (1) and (2) provided baseline accuracy of comprehending grammatically correct and semantically appropriate Russian sentences. On the other hand, accuracy in the semantically implausible conditions (3) and (4) indicated whether the participant relied on semantics (incorrect responses) or syntax (correct responses). For instance, in the sentence *Dima worked with the doctor (Instr*, *masc) of the president (Gen*, *masc)*, *who treat-PART* (Gen, *masc) small children*, the participant’s incorrect answer that the *doctor* treated small children indicated that the participant built a semantically plausible interpretation not supported by morphosyntax, in line with good-enough processing. On the other hand, the correct answer that the *president* treated small children reflected reliance on syntax. Hence, the experimental design implied that reliance on semantics would be reflected in lower comprehension accuracy in semantically implausible sentences.

Semantic plausibility of the stimuli was evaluated in a preliminary online norming study in Google Forms with 188 healthy monolingual participants that were recruited via social networks. The participants were presented with verb phrases corresponding to the participial clause in the experimental sentences, as well as two nouns corresponding to the head and dependent nouns in the genitive noun phrase in the experimental sentences (e.g., *Treat small children*. *Doctor / President*) and were asked to rate which of the two nouns was more plausible as an agent of the action. On a 5-point scale, one noun was placed at 1 and the other at 5, so that, for example, a rating of 1 would indicate that one noun is a much more plausible agent of the action than the other one. Based on the norming study, we selected participles that were rated as highly plausible with one of the nouns in a noun phrase and highly implausible with the other one (mean rating 4.52, range 3.82–5.00, or mean rating 1.44, range 1.03–2.00).

There were three types of filler sentences (N = 128): 1) fillers imitating experimental sentences with high syntactic attachment (N = 22); 2) fillers imitating experimental sentences with low syntactic attachment (N = 22); 3) fillers with a different syntactic structure (N = 84). In fillers, questions never targeted the site of participle attachment (instead, they targeted, adjuncts, subjects, indirect objects, etс.).

The total number of experimental and filler items was equally divided into two sets to be used alternately in the noise and no-noise session. Each stimuli set contained 28 stimuli and 64 filler sentences. In each stimuli set, stimuli were balanced for the grammatical gender of the nouns in the genitive noun phrase: the nouns were feminine in one half of the stimuli and masculine in the other half. Additionally, the stimuli in the two sets were matched for the length of each noun and for the sentence length in syllables. In a Latin square design, four experimental lists for each stimuli set were created.

In the noise session in Experiment 1, stimuli were accompanied with auditory noise. The noise was a three-talker babble made of three popular Russian science podcasts that were merged and overlapped in the version 2.3.3.0 of Audacity(R) recording and editing software [33]. The podcasts covered the following topics: family, cinema, and the Chukchi language (a sociolinguistic podcast about a minority language in Russia). In total, there were nine speakers, three of them were women. Any non-speech sounds (such as music, crackling, rustling) were removed from the podcast. All three tracks were overlapped in a single one with 37 minutes length, which was longer than the whole noise session.

The complete set of stimuli, noise, data, and analysis code are available online at the OSF project page https://osf.io/6dy54/, DOI 10.17605/OSF.IO/6DY54.

#### Procedure

The experiment began with a 9-point calibration and proceeded with instructions presented on the screen. The instruction was followed by five practice trials, after which the experimental trials began.

Each trial started with a drift correction (black fixation dot) at the position of the first letter in the sentence. Once the fixation was detected, a sentence appeared in full. If the fixation detection did not succeed, calibration was repeated. When participants finished reading a sentence and were ready to answer a comprehension question, they fixated the red dot in the lower right-hand corner of the screen. Once the eye tracker detected the fixation, a sentence was replaced by a comprehension question with two response options. The position of the correct option on the screen was pseudorandomized. Participants answered a question by clicking on the selected option. After that, the next trial began.

Sentences were presented in a random order. The lists were assigned to participants pseudo-randomly. Each participant completed the experiment in two sessions: under noise and no-noise conditions. The order of the noise and no-noise session was balanced across the participants. The noise was presented continuously through headphones on the acoustic level of 50 dB during the entire noise session of the experiment, i.e., it accompanied both experimental sentences and comprehension questions. The whole procedure took approximately 50 minutes in total, including a fifteen minutes break between the sessions.

### Analysis

Statistical data analysis included calculation of response accuracy and three eye-movement measures: First Fixation Duration (FFD; the only or the first fixation on a word) and Gaze Duration (GD; sum of all fixations on a word before the first saccade leaving the word), and Total reading Time (TT; sum of all fixations on a word including rereading after leaving the word and returning to it). Eye movement measures were analyzed in two critical regions: the participle and the noun preceding it. All fixations less than 50 milliseconds (resulting in 1% of the data) were deleted from the analysis but no upper cut-off limits were applied. The analysis was conducted with the R system for statistical computing [34] using lme4 package [35].

Response accuracy was analyzed using a generalized mixed-effects model. The model included plausibility (sum-contrast coded; with 1 for implausible condition), noise (sum-contrast coded; with 1 for noise session), and interaction between plausibility and noise. Random effects structure included random intercepts for participants and items as well as by-item random slopes for plausibility and by-participant random slopes for the main effects of plausibility and noise. The full structure of the model was as follows: accuracy ~ plausibility * noise + (1 + plausibility || unique.*item) + (1 + plausibility + noise || subject*.*id)*.

A separate linear mixed-effects model was run for each of the three eye-movement measures. Importantly, the models controlled word form frequency of the participle due to different case endings across conditions: *лечащ****им*** *‘*who treat-PART (Instr, masc)’ / *лечащ****его*** ‘who treat-PART (Gen, masc)’ / *управляющ****им*** ‘who run-PART (Instr, masc)’ / *управляющ****его*** ‘who run-PART (Gen, masc)’ (see Table 1). Word form frequency measures were calculated based on the Russian National Corpus [36]. For the second critical region, controlling for word form frequency was not necessary because the noun preceding the participle had the same case form across conditions, e.g. *президента ‘president (Gen*, *masc)’* in Table 1. Word length was controlled in the models for both regions.

Hence, the models with each eye-movement measure (log-transformed) as an outcome included plausibility (sum-contrast coded; with 1 for implausible condition), noise (sum-contrast coded; with 1 for noise condition), word length (centered), word frequency (centered, for the participle region only), and two two-way interactions (plausibility x noise, plausibility x accuracy). Random effects structure included random intercepts for participants and items. Initially, we also included by-item and by-participant random slopes for the main effects of plausibility and noise and excluded them one by one until the models converged. The final set of models included GD models without random slopes; FFD and TT models for the participle region with by-item random slopes for plausibility and by-participant random slopes for plausibility and noise; FFD model for the noun preceding the participle with by-participant random slopes for plausibility and noise; TT model for the noun preceding the participle with the full random structure. The full possible structure of the models was as follows: log(eyetrackingmeasure_AOI) ~ plausibility * noise + plausibility:*accuracy + length*.*centered + frequency*.*centered + (1 + plausibility + noise || unique*.*item) + (1 + plausibility + noise || subject*.*id)*.

## Results

The overall response accuracy in fillers was 0.95 (by-participant range was 0.84–1.00), which indicated high engagement of participants in the experiment. Thus, no participants were excluded from the analysis. Means and standard deviations for response accuracy and the analyzed eye-movement measures (FFD, GD, TT) are summarized in Table 2.

**Table 2:** Means and SD (in parentheses) of response accuracy and fixation durations at the participle and the noun preceding the participle across conditions (calculated on the raw data).

	Critical region	Accuracy	FFD	GD	TT
		M (SD)	M (SD), ms	M (SD), ms	M (SD), ms
**Plausible, no noise**	Participle	0.74 (0.21)	211 (46)	475 (152)	1000 (575)
	Noun		226 (54)	409 (121)	844 (432)
**Plausible, noise**	Participle	0.72 (0.21)	245 (41)	415 (192)	1010 (559)
	Noun		237 (44)	321 (110)	928 (456)
**Implausible, no noise**	Participle	0.52 (0.28)	223 (48)	492 (167)	1168 (710)
	Noun		222 (58)	399 (104)	948 (506)
**Implausible, noise**	Participle	0.46 (0.27)	237 (40)	432 (202)	1161 (757)
	Noun		234 (37)	324 (96)	981 (514)

### Response accuracy

Response accuracy was lower in the implausible condition compared to the plausible one (Est. = -0.71, SE = 0.12, *z* = -6.07, *p* < 0.001). There was no difference in response accuracy in the noise condition compared to the no-noise condition (Est. = -0.12, SE = 0.08, *z* = -1.47, *p* = 0.14) and no interaction between plausibility and noise (Est. = -0.05, SE = 0.04, *z* = -1.38, *p* = 0.17). In other words, noise did not affect response accuracy in the plausible and implausible conditions differently. The model estimates are presented in Table 3.

**Table 3:** Parameter estimates for the generalized mixed-effects model for the response accuracy.

	ACCURACY			
*Predictors*	*Estimate (Log-Odds)*	*SE*	*z value*	*p*
(Intercept)	0.62	0.08	7.80	**<0.001**
Plausibility	-0.71	0.12	-6.07	**<0.001**
Noise	-0.12	0.08	-1.47	0.141
Plausibility x Noise	-0.05	0.04	-1.38	0.167
N _unique.item_	56			
N _subject.id_	71			
Observations	3950			
Marginal R^2^ / Conditional R^2^	0.109 / 0.311			

#### Eye-movement measures at the participle

**Fig 1:**
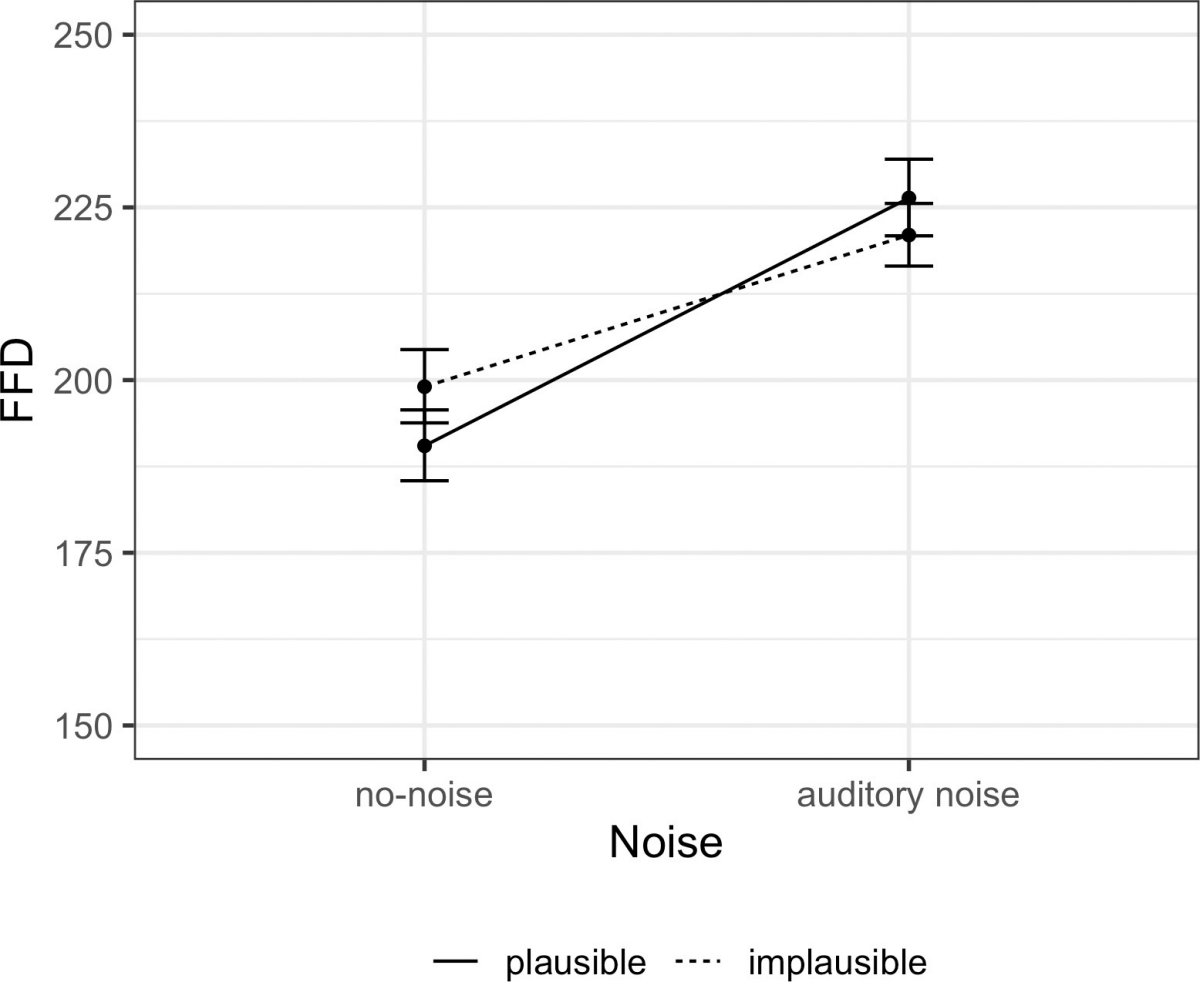
Estimated interaction between noise and plausibility in FFD at the participle.

A series of mixed-effects models revealed that plausibility did not affect either FFD and GD, or TT fixation duration measures (all *ps > 0*.*05*), whereas noise did. In the noise session, participles were read slower in FFD (Est. = 0.07, SE = 0.01, *t* = 7.62, *p* < 0.001) and faster in GD (Est. = -0.09, SE = 0.01, *t* = -10.24, *p* < 0.001). There was an interaction between plausibility and noise in FFD (Est. = -0.02, SE = 0.01, *t* = -2.83 *p* = 0.005), which indicated that in the no-noise condition, implausible sentences were processed slower than plausible (the result of the model with nested comparisons *log(FFD) ~ noise/plausibility + (1 | unique*.*item) + (1 | subject*.*id)*: Est. = 0.04, SE = 0.02, *t* = 2.35, *p* = 0.02), whereas this difference was not found under noise. This interaction can also be seen in Fig 1 (we present means and confidence intervals on a log-odds scale, the data was derived from the model estimates).

Importantly, there was an interaction between plausibility and accuracy in TT (Est. = 0.06, SE = 0.02, *t* = 2.66, *p* = 0.008): longer reading time on participles in implausible sentences increased comprehension (the result of the model with nested comparisons *log(TT) ~ plausibility/accuracy + (1 | unique*.*item) + (1 | subject*.*id)*: Est. = 0.12, SE = 0.03, *t* = 4.08, *p* < 0.001). As expected, longer word forms were processed slower in GD and TT, and more frequent word forms were processed faster in GD and TT. The model estimates are summarized in Table 4.

**Table 4:** Parameter estimates for mixed-effects models for eye-movements measures at the participle.

	FFD				GD				TT			
*Predictors*	*Est*.	*SE*	*t*	*p*	*Est*.	*SE*	*t*	*p*	*Est*.	*SE*	*t*	*p*
(Intercept)	5.35	0.02	240.41	**<0.001**	5.85	0.03	172.04	**<0.001**	6.53	0.06	101.21	**<0.001**
Plausibility	0.01	0.01	1.30	0.194	0.00	0.01	0.15	0.880	0.01	0.02	0.68	0.496
Noise	0.07	0.01	7.62	**<0.001**	-0.09	0.01	-10.24	**<0.001**	0.00	0.02	0.14	0.888
Plausibility x Noise	-0.02	0.01	-2.83	**0.005**	0.01	0.01	0.93	0.351	-0.00	0.01	-0.05	0.962
Plausibility x Accuracy	-0.02	0.01	-1.21	0.228	0.01	0.02	0.63	0.527	0.06	0.02	2.66	**0.008**
Word length	-0.00	0.00	-1.46	0.146	0.03	0.00	6.90	**<0.001**	0.05	0.00	9.19	**<0.001**
Word frequency	-0.01	0.01	-1.55	0.121	-0.03	0.01	-3.20	**0.001**	-0.02	0.01	-2.66	**0.008**
N	56 _unique.item_				56 _unique.item_				56 _unique.item_			
	71 _subject.id_				71 _subject.id_				71 _subject.id_			
Observations	3827				3852				3872			
Marginal R^2^ / Conditional R^2^	0.035 / 0.056				0.039 / 0.220				0.038 / 0.127			

#### Eye-movement measures on the noun preceding the participle

Similarly to the participle region, plausibility did not affect either FFD and GD, or TT fixation duration on the noun preceding the participle (all *ps > 0*.*05*), whereas noise did. In the noise session, the noun preceding the participle was read slower in FFD (Est. = 0.05, SE = 0.01, *t* = 4.19, *p* < 0.001), but faster in GD (Est. = -0.13, SE = 0.01, *t* = -16.76, *p* < 0.001). There was an interaction between plausibility and noise in TT (Est. = -0.02, SE = 0.01, *t* = -2.08, *p* = 0.04), which indicated that in the no-noise condition, implausible sentences were processed slower than plausible (the result of the model with nested comparisons *log(TT) ~ noise/plausibility + (1 | unique*.*item) + (1 | subject*.*id)*: Est. = 0.11, SE = 0.02, *t* = 4.08, *p* < 0.001). Similarly, there was an interaction between plausibility and accuracy in TT (Est. = 0.05, SE = 0.02, *t* = 2.35, *p* = 0.02): longer reading time on nouns preceding the participle in implausible sentences increased comprehension (the result of the model with nested comparisons: Est. = 0.07, SE = 0.03, *t* = 2.33, *p* = 0.02). As expected, longer word forms were processed slower in GD and TT. The model estimates are provided in Table 5.

**Table 5:** Parameter estimates for mixed-effects models for eye-movements measures at the noun preceding the participle.

	FFD				GD				TT			
*Predictors*	*Est*.	*SE*	*t*	*p*	*Est*.	*SE*	*t*	*p*	*Est*.	*SE*	*t*	*p*
(Intercept)	5.32	0.02	254.87	**<0.001**	5.72	0.03	174.75	**<0.001**	6.52	0.06	115.02	**<0.001**
Plausibility	0.01	0.01	0.76	0.445	-0.01	0.01	-1.01	0.311	0.00	0.02	0.25	0.803
Noise	0.05	0.01	4.19	**<0.001**	-0.13	0.01	-16.76	**<0.001**	0.03	0.02	1.59	0.113
Plausibility x Noise	0.00	0.01	0.50	0.620	0.01	0.01	0.81	0.416	-0.02	0.01	-2.08	**0.037**
Plausibility x Accuracy	-0.02	0.02	-1.03	0.304	0.02	0.02	1.29	0.197	0.05	0.02	2.35	**0.019**
Word length	-0.00	0.01	-0.24	0.808	0.04	0.01	3.53	**<0.001**	0.05	0.01	5.74	**<0.001**
N	56 _unique.item_				56 _unique.item_				56 _unique.item_			
	71 _subject.id_				71 _subject.id_				71 _subject.id_			
Observations	3771				3820				3840			
Marginal R^2^ / Conditional R^2^	0.013 / 0.063				0.072 / 0.284				0.043 / 0.102			

### Interim discussion

The results of Experiment 1 showed that auditory noise affected the overall reading speed (the main effect of noise on eye movements that was also previously seen in studies with different auditory noise [3–6]). More specifically, a three-talker background babble led to longer FFD and shorter GD in the critical regions. Probably, participants lengthened their initial fixations to process words more carefully, and as a result, needed less subsequent fixations on a word, which reduced GD. This pattern of eye movements under noise is in line with previous observations of eye-movement behavior under increasing cognitive load in [37, 38].

To remind the reader, auditory noise was expected to affect comprehension due to increased cognitive load and due to modality mismatch (reading vs. listening). However, participants apparently compensated for noise-induced cognitive load with longer first fixations, which enabled them to process sentences efficiently and preserve comprehension rate. The preserved comprehension under auditory noise found in our study is in line with [5], [11 in the bar-type-noise condition], [12], but contradicts [10] and [11 in the non-preferred-music condition].

As predicted by the good-enough account, participants relied overall on semantic relations between words more than on syntactic ones, which is reflected in the main effect of plausibility on reading comprehension. This effect (also seen in [39]) provides evidence of good-enough processing in comprehending Russian sentences. We also found that participants made more mistakes in implausible sentences when they read (too) quickly, but were able to answer questions more accurately when they slowed down.

However, in contrast to our expectations, the readers’ reliance on good-enough processing did not seem to be modulated by noise: participants made more mistakes in implausible compared to plausible sentences regardless of noise. The preserved comprehension under auditory noise was obviously due to changes in the reading pattern (see the interaction between plausibility and noise in FFD for the participle and in TT for the noun preceding the participle). Namely, auditory noise made participants slow down in reading plausible sentences, in comparison with the no-noise conditions (see Tables 4 and 5). We interpret this finding as a detrimental effect of noise on reading plausible sentences due to increased cognitive load.

Taking into account the changes in reading speed coupled with preserved comprehension under noise, we conclude that noise affects online sentence reading but not sentence comprehension. Auditory noise initially impaired reading at the earliest processing stage (seen in FFD increase) but then speeded up reading (seen in shorter GD) without a comprehension cost. Under noise, participants did not rely more on good-enough processing in their interpretations. Apparently, modality mismatch was not detrimental for reading comprehension. Hence, the next question was whether visual noise (being a modality match with a target signal) would affect comprehension accuracy negatively and trigger greater reliance on good-enough processing.

## Experiment 2 (visual noise)

Experiment 2 replicated Experiment 1 in terms of stimuli and procedure with the only difference being the type of noise (visual), which in this case matched the modality of the stimuli.

### Method

#### Participants

Seventy adult Russian monolinguals (30 women; aged 20–40; M_age_ = 23 years; SD = 5.5; mean years of education = 14.5, range 11–22) took part in the experiment with visual noise. None of them participated in Experiment 1 with auditory noise.

#### Apparatus and materials

The apparatus and materials were identical to those of Experiment 1 with the exception for noise. The noise consisted of short Russian idioms and set phrases from 2 to 5 content words in length, for example *vagon i malen’kaya telezhka* (literally: *a carriage and a small trolley*, idiomatic: *tons and tons of something*), *delat’ iz mukhi slona* (literally: *to make an elephant out of a fly*, idiomatic: *to make mountains out of molehills*) etc. In total, 400 frequent and well-known Russian idioms were selected. The complete list of idioms is available online at the OSF project page https://osf.io/6dy54/, DOI 10.17605/OSF.IO/6DY54.

#### Procedure

**Fig 2:**
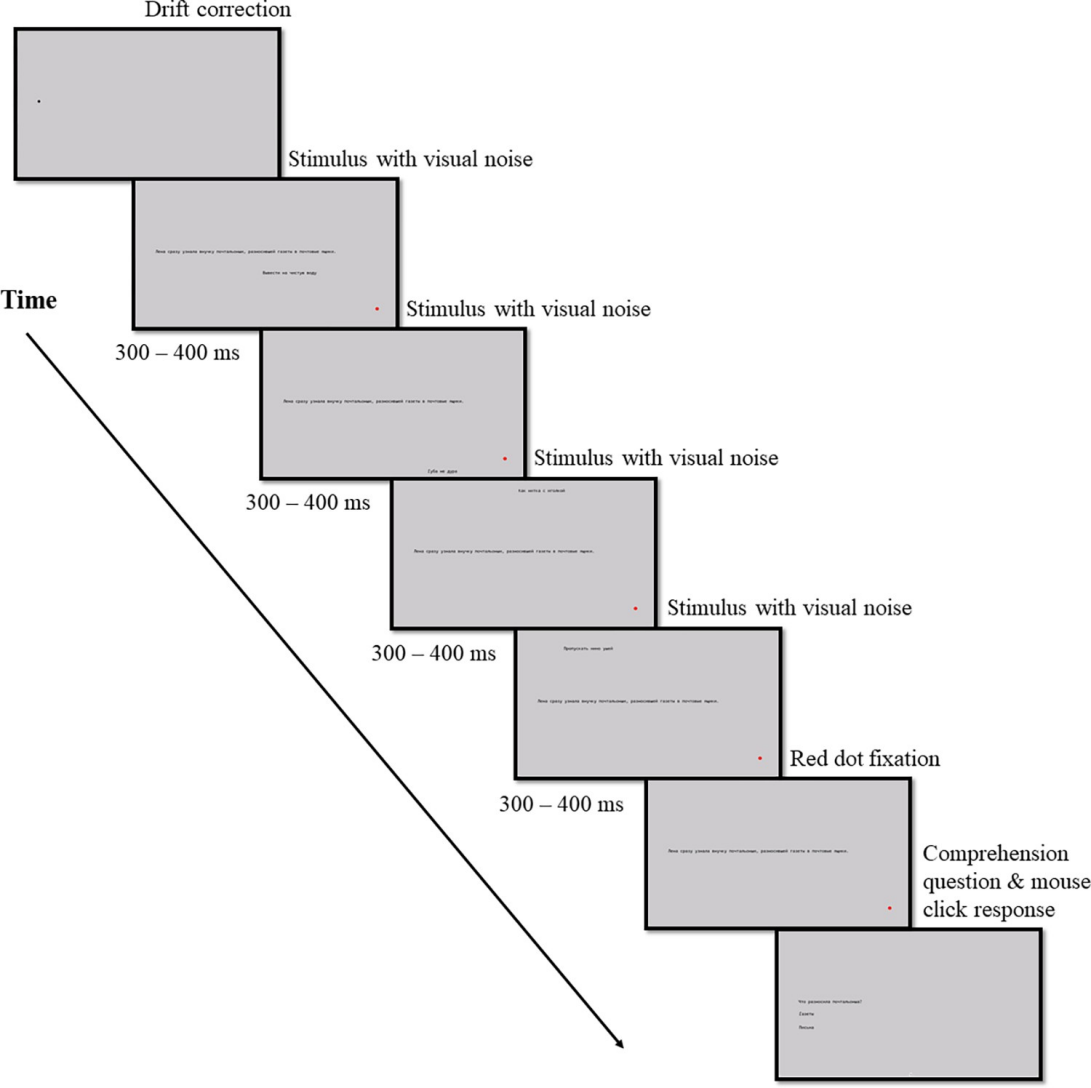
An experimental trial with visual noise.

The experimental procedure was similar to that of Experiment 1. However, instead of auditory noise, visual noise accompanied the presentation of experimental sentences (but not comprehension questions). For each experimental sentence, 3–4 randomly chosen idioms appeared consecutively at random positions on the screen around the experimental sentence and remained for 300–400 ms each. Visual noise never overlapped with the experimental sentence. A demonstration of a trial with visual noise is provided below in Fig 2.

### Analysis and results

Statistical data analysis was similar to that of Experiment 1. We also deleted from the analysis all fixations shorter than 50 milliseconds (resulting in 1% of the data loss). The only difference was in the random structure of linear mixed-effects models: in the experiment with visual noise, FFD and GD models included by-item and by-participant random slopes for the main effects of plausibility and noise, whereas TT models included only by-participant random slopes for plausibility and noise. The overall response accuracy in fillers was 0.95 (by-participant range was 0.88–1.00). No participants were excluded from the analysis. Means and standard deviations for response accuracy and eye-movement measures (FFD, GD, TT) are summarized in Table 6. The supplementary analysis of the number of fixations and fixation durations on short idioms and set phrases is available online: https://osf.io/6dy54/.

**Table 6:** Means and SD (in parentheses) of response accuracy and fixation durations at the participle and the noun preceding the participle across conditions (calculated on the raw data).

	Critical region	Accuracy	FFD	GD	TT
		M (SD)	M (SD), ms	M (SD), ms	M (SD), ms
**Plausible, no noise**	Participle	0.73 (0.22)	254 (41)	440 (174)	913 (620)
	Noun		244 (48)	323 (104)	777 (437)
**Plausible, noise**	Participle	0.75 (0.23)	243 (41)	411 (179)	833 (595)
	Noun		243 (45)	315 (119)	728 (425)
**Implausible, no noise**	Participle	0.43 (0.26)	251(39)	446 (187)	977 (694)
	Noun		241 (44)	323 (108)	846 (502)
**Implausible, noise**	Participle	0.42 (0.30)	250 (42)	427 (191)	944 (766)
	Noun		247 (48)	325 (116)	826 (704)

### Response accuracy

**Fig 3:**
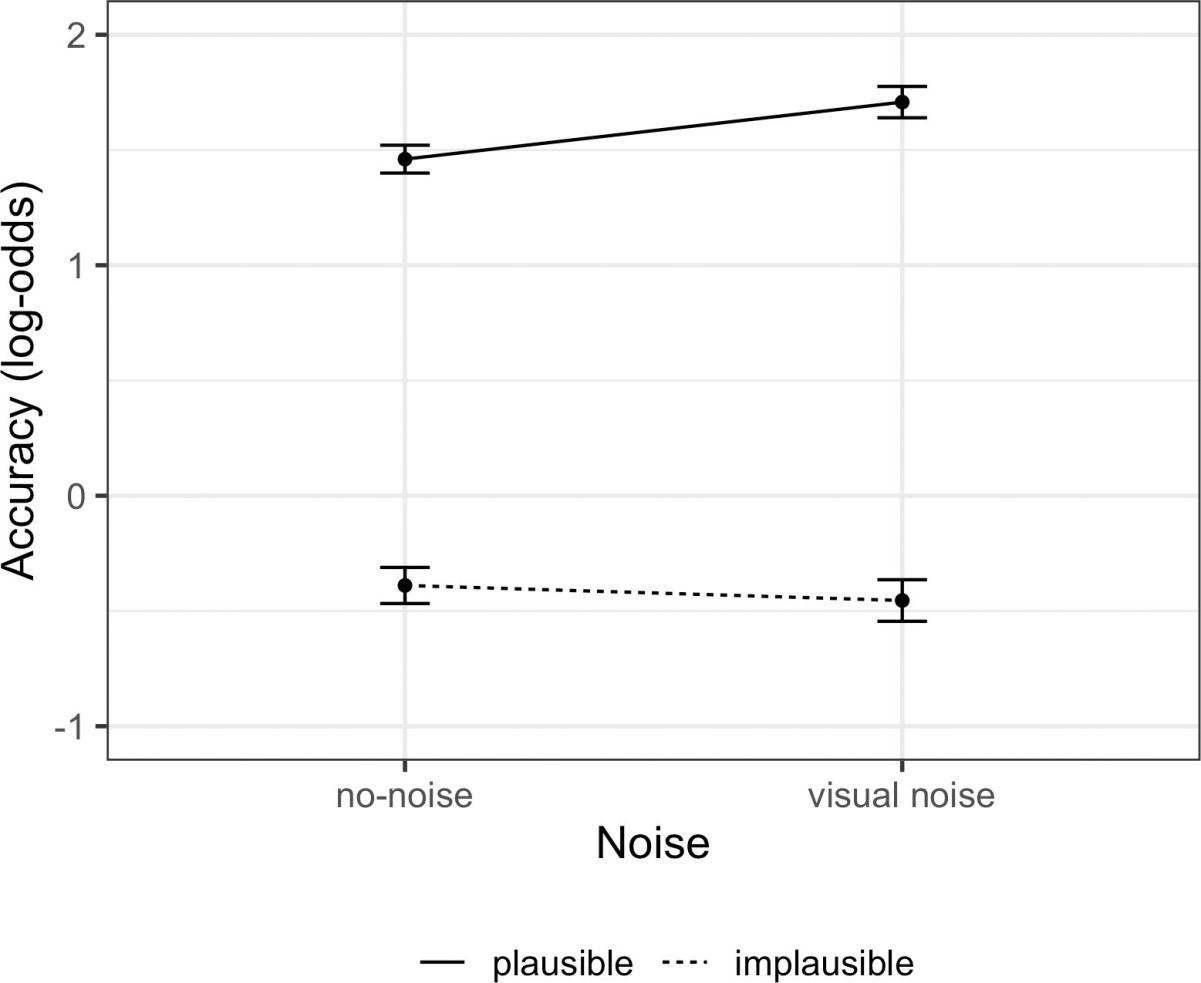
Estimated interaction between noise and plausibility for accuracy in Experiment 2 with visual noise. The figure shows partial effects from the mixed-effects model.

Statistical analysis of response accuracy with a generalized mixed-effects model revealed lower response accuracy in implausible sentences (Est. = -0.92, SE = 0.14, *z* = -6.71, *p* < 0.001). The model estimates are presented in Table 7. Interestingly, although noise did not affect the overall response accuracy, there was an interaction between plausibility and noise (Est. = -0.08, SE = 0.04, *z* = -1.97, *p* = 0.048), which indicated that in the presence of visual noise, plausible sentences tended to be processed more accurately, whereas implausible sentences tended to be processed less accurately, see Fig 3. However, in the model with nested comparisons, neither of these tendencies reached significance (*accuracy ~ plausibility/noise + (1 | unique*.*item) + (1 | subject*.*id)*; in the plausible condition: Est. = 0.13, SE = 0.10, *z* = 1.32, *p* = 0.19; in the implausible condition: Est. = -0.004, SE = 0.09, *z* = -0.04, *p* = 0.97).

**Table 7:** Parameter estimates for the generalized mixed-effects model for the response accuracy.

	ACCURACY			
*Predictors*	*Estimates (Log-Odds)*	*SE*	*z*	*p*
(Intercept)	0.54	0.09	6.25	**<0.001**
Plausibility	-0.92	0.14	-6.71	**<0.001**
Noise	0.04	0.08	0.57	0.571
Plausibility x Noise	-0.08	0.04	-1.97	**0.048**
N _unique.item_	56			
N _subject.id_	70			
Observations	4255			
Marginal R^2^ / Conditional R^2^	0.160 / 0.384			

#### Eye-movement measures at the participle

Similarly to the Experiment 1, a series of mixed-effects models revealed that plausibility did not affect either FFD and GD or TT fixation duration measures (all *ps > 0*.*05*), whereas noise did, though not in the way expected. Surprisingly, in the noise condition, the critical participle was read faster in GD (Est. = -0.03, SE = 0.01, *t* = -2.43, *p* = 0.015) and TT (Est. = -0.04, SE = 0.02, *t* = -2.42, *p* = 0.015). No interaction between noise and plausibility was found (all *ps > 0*.*05*). Importantly, there was an interaction between plausibility and accuracy in TT similar to the one in Experiment 1 (Est. = 0.06, SE = 0.02, *t* = 2.85, *p* = 0.004): longer reading times on participles in implausible sentences led to correct responses (the result of the model with nested comparisons *log(TT) ~ plausibility/accuracy + (1 | unique*.*item) + (1 | subject*.*id)*: Est. = 0.11, SE = 0.03, *t* = 4.05, *p* < 0.001). As expected, more frequent word forms were processed faster in all measures, and longer word forms were processed slower in GD and TT, though faster in FFD. The model estimates are summarized in Table 8.

**Table 8:** Parameter estimates for mixed-effects models for eye-movements measures on the participle.

	FFD				GD				TT			
*Predictors*	*Est*.	*SE*	*t*	*p*	*Est*.	*SE*	*t*	*p*	*Est*.	*SE*	*t*	*p*
(Intercept)	5.47	0.02	295.21	**<0.001**	5.79	0.04	134.20	**<0.001**	6.38	0.07	92.25	**<0.001**
Plausibility	0.01	0.01	1.41	0.160	-0.00	0.01	-0.03	0.977	-0.01	0.02	-0.69	0.492
Noise	-0.01	0.01	-1.81	0.070	-0.03	0.01	-2.43	**0.015**	-0.04	0.02	-2.42	**0.015**
Plausibility x Noise	0.01	0.01	1.66	0.098	0.00	0.01	0.10	0.924	0.01	0.01	1.21	0.226
Plausibility x Accuracy	-0.01	0.01	-1.22	0.222	0.02	0.02	0.85	0.396	0.06	0.02	2.85	**0.004**
Word length	-0.01	0.00	-2.80	**0.005**	0.03	0.00	7.18	**<0.001**	0.04	0.00	7.87	**<0.001**
Word frequency	-0.01	0.00	-3.08	**0.002**	-0.03	0.01	-3.49	**<0.001**	-0.03	0.01	-3.38	**0.001**
N	56 _unique.item_				56 _unique.item_				56 _unique.item_			
	70 _subject.id_				70 _subject.id_				70 _subject.id_			
Observations	4196				4200				4207			
Marginal R^2^ / Conditional R^2^	0.008 / 0.011				0.027 / 0.060				0.033 / 0.105			

#### Eye-movement measures on the noun preceding the participle

Similarly to the participle region, plausibility did not affect either early FFD and GD, or late TT fixation duration measures on the noun preceding the participle (all *ps > 0*.*05*). We found no effect of noise and no interactions (all *ps > 0*.*05*). As expected, longer word forms were processed slower in both GD and TT. The model estimates are presented in Table 9.

**Table 9:** Parameter estimates for mixed-effects models for eye-movements measures at the noun preceding the participle.

	FFD				GD				TT			
*Predictors*	*Est*.	*SE*	*t*	*p*	*Est*.	*SE*	*t*	*p*	*Est*.	*SE*	*t*	*p*
(Intercept)	5.41	0.02	269.64	**<0.001**	5.59	0.03	168.78	**<0.001**	6.34	0.06	99.06	**<0.001**
Plausibility	0.02	0.01	1.59	0.111	0.02	0.01	1.38	0.167	0.01	0.01	0.80	0.422
Noise	0.01	0.01	0.93	0.350	-0.00	0.01	-0.24	0.813	-0.03	0.02	-1.52	0.129
Plausibility x Noise	0.00	0.01	0.84	0.402	0.01	0.01	1.84	0.065	-0.00	0.01	-0.38	0.703
Plausibility x Accuracy	-0.02	0.01	-1.26	0.207	-0.01	0.02	-0.83	0.407	0.02	0.02	1.00	0.318
Word length	0.00	0.00	1.03	0.301	0.04	0.01	6.31	**<0.001**	0.04	0.01	4.25	**<0.001**
N	56 _unique.item_				56 _unique.item_				56 _unique.item_			
	70 _subject.id_				70 _subject.id_				70 _subject.id_			
Observations	4264				4273				4283			
Marginal R^2^ / Conditional R^2^	0.002 / 0.011				0.035 / 0.060				0.028 / 0.092			

### Interim discussion

The results of Experiment 2 showed changes in overall reading speed under visual noise: participles were read faster (seen in GD and TT decrease). This speedup does not match previous findings: Longer fixations under visual noise were previously observed with blurred script in [7, 8], and with short phrases appearing on the screen together with the target sentences in [9]. We interpret this speedup as driven by participants’ desire to complete the reading task as fast as they can, presumably because visual noise makes reading uncomfortable.

At the same time, we found no main effect of noise on comprehension accuracy. Participants managed to read sentences under noise even faster, preserving a high comprehension rate. At present, it is unclear why visual noise did not disrupt comprehension. Potential explanations, such as easy adaptation to modality-matching noise (just like we adapt to pop-up notifications and advertisement posts on smartphones and computer screens), or to the noise that does not overlap with the signal, must remain speculative at present.

Furthermore, Experiment 2 replicated the main effect of plausibility on comprehension accuracy and the interaction between plausibility and accuracy (seen in the total reading time of the participle). In implausible sentences, we found that the participants made more mistakes when they read quickly, but were able to answer questions more accurately when they slowed down. This indicated that participants who read quickly relied on semantics rather than syntax while reading semantically implausible Russian sentences, just in line with the good-enough processing model. Taking into account that experimental material was the same in both experiments, these findings present a replication of the effect across two participant samples and provide reliable evidence of good-enough processing when reading grammatically complex Russian sentences (see the comparison of response accuracy between the two experiments at https://osf.io/6dy54/).

Finally, the participants’ reliance on good-enough processing did not increase under visual noise: we found no interaction between plausibility and noise in reading speed. Although we found an interaction between plausibility and noise in comprehension accuracy, the difference in accuracy in nested comparisons was not significant.

## General discussion

The present study aimed to investigate whether auditory and visual noise would increase reliance on semantics during reading, as predicted by the good-enough processing and noisy-channel accounts [13–15]. Specifically, we conducted two eye-tracking experiments testing whether Russian-speaking adults would misinterpret semantically implausible sentences more often in the presence of noise and whether noise would affect their reading patterns. The main findings of the study are as follows: (1) Russian-speaking readers generally relied on semantics and misinterpreted implausible sentences, as predicted by the good-enough processing account. (2) Neither auditory nor visual noise increased the readers’ reliance on good-enough processing. (3) Both auditory and visual noise affected reading patterns: auditory noise increased FFD, whereas both auditory and visual noise decreased GD.

The first finding, that semantic plausibility influenced comprehension accuracy in both experiments indicates that Russian-speaking readers quite often (40% accuracy) interpreted sentences according to their real-world knowledge and semantic relations between words instead of relying on syntactic roles, which resulted in more comprehension errors in the implausible conditions. This result is in line with the predictions of the good-enough processing model [19, 20]. Importantly, in both experiments, we observed that longer total reading time was associated with an accuracy increase for implausible sentences. This is predicted by the good-enough processing model [40] and indicates that good-enough, semantically-based processing is faster than syntactically-based algorithmic processing.

### Reliance on good-enough processing under noise

The second finding was that neither auditory nor visual noise increased the readers’ reliance on good-enough processing. We suggest several explanations for this. First, the discrepancy between our results, the predictions of the noisy-channel account and previous experimental studies may be due to the noise saliency and the degree of distraction from the target signal. We did not measure or manipulate the noise saliency, and participants apparently inhibited both types of noise quite well. Second, our study was among the first ones to investigate reliance on semantics using semantically (im)plausible sentences under external noise, as opposed to degraded quality of the target signal, such as blurred script or certain font types in [4, 5]. In our experiments, neither auditory nor visual noise overlapped with the target stimuli: in Experiment 1, a three-talker babble was in the auditory modality, whereas the target stimuli were in the written modality; in Experiment 2, idioms appeared for a short time on the screen around target sentences without physically overlapping the target input.

At the same time, we found an interaction between plausibility and noise in reading times under auditory noise. Without noise, readers processed critical words in plausible sentences faster than in implausible ones, but this effect disappeared in the presence of auditory noise. This might indicate that under noise, simpler semantically plausible sentences were processed as effortfully as more complex semantically implausible sentences. These results are unlike the findings in [4], where the syntactic complexity effect was not modulated by auditory noise.

### Main effects of auditory noise

The lack of the main effect of background babble on sentence comprehension accuracy was in line with previous studies testing similar types of auditory noise. Namely, Johansson and colleagues [11] did not find a significant difference in comprehension accuracy during reading under the noise typical for cafés, nor did Vasilev and colleagues [5] and Yan and colleagues [6] who investigated the influence of native and foreign background speech on reading. Despite methodological differences, e.g., different noise saliency, instructions, and noise presentation, our results are consistent with the previous studies. Altogether, this means that background auditory noise with human speech tends to be easy to suppress for readers.

The main effect of auditory noise on reading speed in our study partially aligns with some earlier findings of the studies investigating meaningful speech as auditory noise (e.g., slow-down effect on eye-movements in [3, 4]). To remind the reader, we observed a slow-down in earlier processing (shown in FFD increase) on both participle and the preceding noun, as well as a speed-up in later processing of both critical regions (shown in GD decrease). Several studies of intelligible (e.g., [3–5]) and unintelligible background speech [6] found an increased rereading time and more regressions under noise, which indicated overall difficulties in processing. We suggest that the overall pattern of fixation durations on the noun and the participle in the presence of noise indicates that initially, readers required more time for lexical activation due to the increased cognitive load, but then they sped up to pick up their normal processing speed. This enabled them to process sentences under noise with comprehension accuracy similar to that of the no-noise conditions.

### Main effects of visual noise

The lack of the main effect of visual noise on sentence comprehension is in line with a previous study using similar visual noise [9]. Visual noise of this kind seems easy to suppress thanks to modality match and no overlap with the target sentences. Whether no comprehension effect was observed due to the participants’ great ability to noise inhibition, or due to low noise saliency remains an open question.

Interestingly though, our results for eye fixation durations under visual noise contradict the findings with blurred script in [7, 8] and with short phrases appearing on the screen together with target sentences in [9]. Whereas those studies found longer fixations under noise, we observed shorter fixations. Our participants accelerated during reading the participles. This surprising effect may be due to the participants’ intention to complete the task sooner and avoid annoying visual noise.

### Limitations and conclusions

We have to acknowledge several limitations of our study. First, auditory and visual noise was not matched in saliency, which could explain their different effects on reading. We cannot distinguish whether the effects of visual and auditory noise differ due to their modality or salience. Another experiment with a different type of noise, more salient and matched for salience across modalities, could shed light on this. In addition, stronger noise might drive increased reliance on good-enough processing, which was not found in our experiments.

Second, our experimental design did not allow us to disentangle between two possible explanations of the plausibility-by-accuracy interaction in TT. According to the one explanation, a faster reading speed of implausible sentences was associated with decreased accuracy. The alternative explanation implies that slower reading speed was associated with accuracy increase. Possibly, correct responses in implausible sentences took longer because the reader detected the semantic mismatch, and needed more time to build a representation of an implausible event. So, in the absence of a baseline condition, for which plausibility does not play any role, we cannot unambiguously explain our results as the slow-down for correct responses and not as a speed-up for incorrect responses. Comparing plausible and implausible conditions to the baseline for which semantic plausibility does not apply remains a promising venue for further investigation.

To summarize, we found evidence for the reliance on good-enough sentence processing during reading in Russian. Having said that, we could not confirm the predictions that noise increases reliance on semantics. This does not invalidate the noisy-channel and good-enough processing models, and rather requires further research on this topic. Crucially, auditory and visual noise affected reading patterns differently: in the presence of auditory noise, readers were first distracted and slowed down at the earliest, but they sped up later, whereas under visual noise readers accelerated their processing.
